# Filtration Properties of Auxetics with Rotating Rigid Units

**DOI:** 10.3390/ma11050725

**Published:** 2018-05-03

**Authors:** Daphne Attard, Aaron R. Casha, Joseph N. Grima

**Affiliations:** 1 Metamaterials Unit, Faculty of Science, University of Malta, MSD2080 Msida, Malta; daphne.attard@um.edu.mt; 2 Faculty of Medicine, University of Malta, MSD2080 Msida, Malta; aaron.casha@um.edu.mt; 3 Department of Chemistry, Faculty of Science, University of Malta, MSD2080 Msida, Malta

**Keywords:** auxetic, negative Poisson’s ratio, porosity, filters

## Abstract

Auxetic structures and materials expand laterally when stretched. It has been argued that this property could be applied in the design of smart filters with tunable sieving properties. This work analyses the filtration properties of a class of auxetic structures which achieve their auxeticity through a rotating rigid unit mechanism, an archetypal mechanism known to be responsible for this behavior in a number of crystalline materials. In particular, mathematical expressions are derived for the space coverage of networks constructed from a variety of quadrilaterals, as well as the pore radius. The latter is indicative of the particle size that can pass through when the particle dimension is comparable to the pore size, whereas the space coverage is indicative of the rate of flow when the particles are of a much smaller dimension than the pore size. The expressions suggest that these systems offer a wide range of pore sizes and space coverages, both of which can be controlled through the way that the units are connected to each other, their shape and the angle between them.

## 1. Introduction

When a material is stretched it changes shape. The changes in dimensions that occur in the *Ox_i_*-*Ox_j_* plane upon uniaxial stretching are quantified by Poisson’s ratio (*ν_ij_*) which is mathematically defined by:

(1) $$\nu_{ij}=-\frac{\varepsilon_{j}}{\varepsilon_{i}}$$

where *ε_i_* is the strain along the loading direction and *ε_j_* is the strain along a perpendicular direction. For three dimensional isotropic materials, Poisson’s ratio may range between −1 ≤ *ν* ≤ +0.5 [1] while for two dimensional isotropic materials, it may range between −1 ≤ *ν* ≤ +1 [2], with no upper or lower bounds for anisotropic materials. Typical materials have a positive Poisson’s ratio and contract laterally when a uniaxial tensile load is applied whereas materials with a negative Poisson’s ratio, more commonly referred to as auxetic [3], expand laterally. This behaviour has been reported in simple mechanical [4,5,6] and thermodynamic [7] models in the 1980s. Earlier, there were some isolated reports of systems with negative Poisson’s ratios including ferromagnetic films [8] and fcc crystals [9]. To date there is a vast range of known auxetic systems that span over a very wide length scale from the nano up to the macro level [10,11,12], including amongst others, minerals [13,14,15,16,17], cubic materials [9,18,19,20], carbon-based systems [3,21,22,23,24,25,26], systems of hard bodies [7,27,28,29], foams [30,31,32,33,34,35], polymers [36,37,38,39], composites [40,41,42,43,44,45,46,47,48,49] and textiles [50,51,52,53,54,55,56].

The rotating rigid unit mechanism remains one of the archetypal mechanisms known to contribute towards auxeticity [57,58,59,60,61,62]. In their most ideal form, systems that achieve negative Poisson’s ratios based on this mechanism consist of a network of rigid polygons or polyhedral units that are connected at their vertices. Uniaxial stretching of the network causes these units to rotate relative to each other to open up pores within the structure, an effect which could possibly lead to expansion in the transverse direction and hence auxeticity.

One of the first to be rigorously studied in this class of structures was the rotating squares geometry [57]. It has been shown that systems based on squares always exhibit an isotropic in-plane Poisson’s ratio of −1. In later studies, this concept was generalised and extended further to include other types of polygons, including rotating triangles, rectangles, rhombi and parallelograms [58,59,60,61,62], as well as 3D polyhedra such as cubes, cuboids and tetrahedra [63,64,65,66]. These studies have generally shown that by changing the shape, the mode of connection and the angle between the rotating rigid units, a wide range of Poisson’s ratio from highly negative to highly positive could be achieved.

It has also been repeatedly suggested that this mechanism could play a significant role in the auxetic behavior demonstrated by a number of materials including silicates [13,16,67,68,69,70], zeolites [14,71] and foams [72,73]. Some of these rotating rigid unit models have also been incorporated in the design of oesophageal stents [74,75], sports shoes and garments [76,77,78], textiles [53], perforated sheets [79,80], and composites [81,82]. To mention a few examples, in their work Hu et al. [53] incorporated a rotating rectangular [83] motif in a fabric produced through flat knitting technology. Nike Inc. (Beaverton, OR, US) [76] developed auxetic soles that mimic a rotating triangle mechanism [59] to achieve an auxetic effect. The expansion of the sole provides improved traction when the wearer engages in activities such as running that puts the sole under tension. Under Armour Inc. (Baltimore, MD, US) has also put forward designs of garments and apparel incorporating auxetic geometries [77], including ones based on rotating units, for improved conformability to provide the wearer with a comfort fit. In this respect, there has also been development in computational methods for the interactive 3D design of garments [78] based on perforated flat sheets that mimic rotating triangles [80].

In addition to these applications, systems made from rotating rigid units also have potential to be used in filtration. Although structures like re-entrant honeycombs have been rigorously studied vis-à-vis filtration [84,85,86,87,88], the properties of rotating rigid unit systems relating to porosity remain largely unexplored. In view of this, the work presented here is a comparative study of the porosity and Poisson’s ratios of networks made from various rotating rigid unit systems, namely, rotating squares, rectangles, rhombi and parallelograms.

## 2. The Models

**Figure 1:**
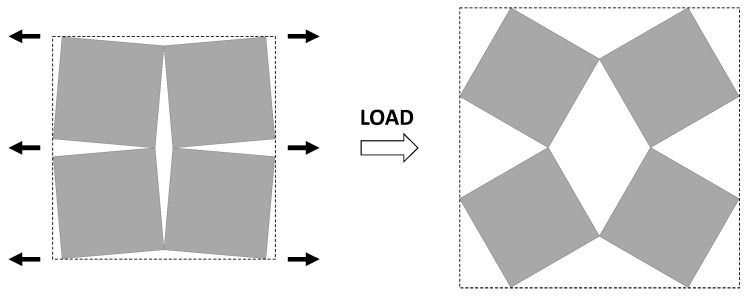
A network of rigid squares connected at their corners through flexible hinges expands when uniaxially stretched, preserving the aspect ratio in the process.

As illustrated in Figure 1, systems made from perfectly rigid squares connected together at their vertices, and which rotate relative to each other when uniaxially stretched, have the ability to expand laterally in a manner which preserves the aspect ratio. This behavior results in a constant Poisson’s ratio of -1 irrespective of the size of the square, the direction of loading and the angles between the squares. Whilst this has the advantage that this system is very robust vis-à-vis its auxetic potential, it has the limitation that it cannot be modified easily so as to fine-tune its macroscopic properties to make it achieve particular values of Poisson’s ratio. This limitation may be overcome if one had to replace the squares (which only need one geometric variable to describe their shape and size, their side length *a*) by more generic quadrilaterals having lower symmetry such as rectangles (which could be considered as elongated squares, the shape and size of which are defined by two variables *a* × *b*), rhombi (which could be considered as sheared squares, the shape and size of which are defined by two variables: the side length *a* and the internal angle *φ*) or parallelograms (which could be considered as elongated and sheared squares, the shape and size of which are defined by three variables: the side lengths *a*, *b* and the internal angle *φ*). Furthermore, it has already been reported in previous work that, for networks based on rectangles, there are two possible types of connectivity [62]. In the first type, referred to as Type I, adjacent rectangles have their smaller sides connected to each other. In the second type, referred to as Type II, each rectangle has its smaller sides connected to the longer side of adjacent rectangles. Similarly, there are also two types of connectivity for networks based on rhombi—the Type *α* and Type *β* [60]. In Type *α*, the rhombi are connected such that the smaller angles of one rhombus are connected to the larger angles of adjacent rhombi. In Type *β*, the smaller angles of the rhombi are connected to smaller angles of adjacent rhombi. For parallelograms, which share characteristics with both rectangles and rhombi, there are four types of connectivity: Type I*α*, Type I*β*, Type II*α* and Type II*β*.

### 2.1. Geometrical Analysis

An analysis of the pore shape of these networks clearly suggests that any of the quadrilaterals which have a Type I connectivity or are rhombic in shape (Type I rectangles, Type *α* and Type *β* rhombi, Type I*α* and Type I*β* parallelograms) have rhombic pores while those that have a Type II connectivity (Type II rectangles, Type II*α* and Type II*β* parallelograms) have parallelogram-shaped pores. Type *α* connectivity or rectangular units (Type I and Type II rectangles, Type *α* rhombi, Type Iα and Type II*α* parallelograms) result in pores that are of similar shape i.e., all pores have equal internal angles, while Type *β* connectivity (Type *β* rhombi, Type I*β* and Type II*β* parallelograms) results in two sets of pores that have different internal angles at any given instant. Moreover, networks derivable from Type *α* connectivity are also space filling as opposed to networks with a Type *β* connectivity which even in their fully closed conformation still have space between the units. On the other hand, Type II*α* parallelograms and systems derivable from them (i.e., squares, Type II rectangles and Type *α* rhombi) have pores that are identical in shape. This is summarised in Table 1.

### 2.2. Pore Size and Surface Coverage

**Figure 2:**
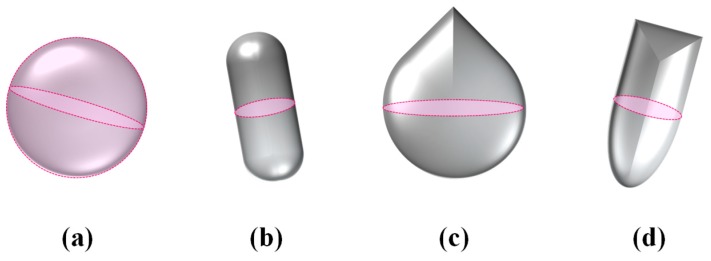
Examples of rigid particles having (**a**) spherical (**b**) pill-box (**c**) drop and (**d**) bullet shapes and the corresponding circular cross-sectional area (indicated by the dashed line) of radius *r*.

**Figure 3:**
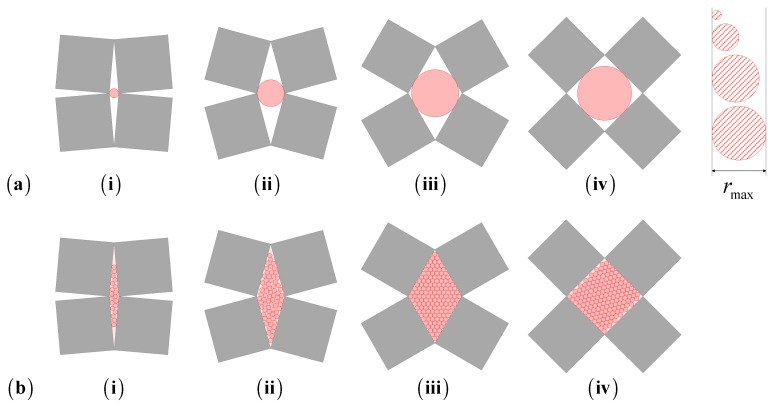
Comparison of different particles sizes as a rotating rigid unit system is stretched (i to iv), illustrating the notion of (**a**) pore size and (**b**) space coverage.

Characterization of a system in terms of its filtration and/or sieving properties requires an analysis that looks at the shape and size of the pores and also their density. In particular, for any porous system, one can define the pore size in terms of the radius of the largest particles that can pass through. This analysis is by no means trivial as it depends on both the size and shape of the pore, and also on the shape and size of the mobile particles that have to be filtered (see Figure 2). In fact, two scenarios need to be considered: one where the mobile particles are of dimensions which are comparable to the size of the pores (Figure 3a) and one where the particles are much smaller than the pores (Figure 3b). In the case of the former, the problem to be studied is that of assessing which particles can physically pass through the pores and which will not. To simplify the analysis, it shall be assumed that the mobile particles are rigid and can all be modelled as units which have a circular cross-section with radius *r*. Through such a description, a parameter *r*_max_ can also be defined as the threshold which defines the sizes of particles that can be filtered: anything smaller than this radius will pass through the filter while anything larger would be retained. This parameter depends, amongst other things, on the degree of aperture of the systems.

In the case where the mobile particles are much smaller than the pores (e.g., a fluid passing through mm sized pores), it would be more appropriate to look at the rate at which the process occurs, in which case one should look at a different parameter, namely, the space coverage, which for the 2D systems analysed here is given mathematically by:

(2) $$\mathrm{Space}\;\mathrm{coverage}=\frac{\mathrm{Area}\;\mathrm{of}\;\mathrm{pores}}{\mathrm{Unit}\;\mathrm{cell}\;\mathrm{area}}$$

For example, in the case of squares of side length *a*, the equation for the space coverage as a function of the angle *θ* between the squares becomes:

(3) $$\mathrm{Space}\;\mathrm{coverage}=\frac{\sin\left(\theta \right)}{\sin\left(\theta \right)+1}$$

Note that this ‘space coverage’ parameter quantifies the fraction of the area that the pores cover out of the whole structure and is a quantity which in practice affects the rate of flow across the filter for particles whose radius is much smaller than the pore size. Once again, space coverage is a parameter which depends, amongst other things, on the degree of aperture of the systems; the more open the structure the higher the pore coverage is and hence the higher the rate of flow of these particles.

### 2.3. Mathematical Modelling

In an attempt to obtain a better insight, both the pore size and the space coverage (assuming an infinite network) were derived and expressed in terms of the measurable parameters *a* and *b* (the side of the quadrilateral), *φ* (the internal angle of the quadrilateral) and *θ* (the degree of openness of the pores) so that by varying *θ*, the size of the filtered particles can be controlled. Here, it should be noted that under the assumption that the squares, rhombi, rectangles or parallelograms are rigid, for a given structure the terms *a*, *b* and *φ* can be treated as constants, i.e., the pore size and the space coverage are functions of the single variable *θ*, which can be made to change through, for example, uniaxial loading.

## 3. Results and Discussion

The expressions for the space coverage and pore radius are summarized in Table 2, together with plots for their variation with the parameter *θ* (the degree of openness of the systems) and plots of Poisson’s ratio as obtained from models developed in previous work derived at infinitesimal strains [57,60,61,62,83]. These plots clearly show that the space coverage and pore radius of the systems under consideration (i) have different profiles which depend on the system being modelled; and, (ii) may be changed in a significant manner by changing the parameter *θ* (i.e., for example, as a result of uniaxial loading), thus highlighting the versatility of these systems.

A feature that is immediately apparent, by visually examining the structures and considering their space filling properties (see Table 1), is the ability of most of the networks (all except Type *β* systems) to have a zero pore size when the system is in its most compact form. In contrast to this, irrespective of the angle between the rigid quadrilaterals, the Type *β* systems, which are not space-filling, always have open pores (a void space between the quadrilaterals).

Moreover, from the unit cells provided in Table 2, networks based on squares, rectangles, rhombi, Type I*β* parallelograms and Type IIα parallelograms can all be represented by a rectangular unit cell. This unit cell remains rectangular even during deformation, a property which is very convenient if such systems are to be used in filtration applications. Although the Type II*β* parallelograms have a parallelogramic unit cell, their internal angle also remains unchanged during deformation. This is in contrast to the Type Iα for which the unit cell is a parallelogram which shears throughout the deformation process, a feature which might make it difficult to implement in practical applications.

Also interesting is the fact that, although it may be intuitive to associate the most open configurations (i.e., the highest space coverage for a given system) with the largest allowable pore radius, this may not always be true. In fact, this is only applicable in systems with a Type *α* connectivity. Using the expressions for the space coverage and pore radius in Table 2, it is easy to show that for Type *α* systems both space coverage and pore radius are at a maximum at *θ* = 90°, whereas for systems with Type *β* connectivity, the maximum pore radius occurs at *θ* = max(π/2, 2*φ* – π/2 while the maximum space coverage occurs at *θ* = *φ* in Type *β* rhombi and Type II*β* parallelograms and θ =*φ* + arctan((*a*^2^ − *b*^2^)/(*a*^2^ + *b*^2^)tan(φ)) in Type I*β* parallelograms. This means that for a given system, a higher space coverage does not necessarily mean a better ability at filtering larger particles. This is also true if one compares the different systems with each other. For example, if Type *α* rhombi are compared with squares, it is apparent that although the Type *α* rhombi have a larger degree of space coverage than the rotating squares, the maximum pore radius is the same in both cases, owing to the fact that in both networks the pores have the same shape so that the difference in pore coverage is merely reflected as a difference in the density of the filter (Type *α* rhombi have a smaller density which becomes smaller as *φ* → 0).

Furthermore, it is evident that systems with a Type *α* connectivity offer a larger range of pore radii, anywhere between 0 in their closed configuration and *a*/2 for Type I connectivity or *b*/2 for Type II connectivity. This is in contrast to what is observed with Type *β* systems, for which the range of pore radius is much smaller and also highly dependent on *φ*. This is because the Type *β* systems are not space-filling and irrespective of the value of *θ* there will always be some void space between the rigid units. This empty space increases as *φ* deviates from 90°. In other words Type *β* systems are always porous even in their most closed configuration as opposed to Type *α* networks, unless *φ* = 90°. In fact, it can be easily shown that for a simple *β* system, such as the Type II*β* parallelograms, for cases where *a* > *b*, the maximum pore radius is *b*/2 (at *θ* = 90°, 2*φ* − 90°) when *φ* ≥ 45° and in cases when *φ* < 45° the maximum pore radius is *b*/2 at *θ* = 90°. On the other hand, the smallest pore radius that the Type II*β* system can afford is of ⅟_2_*b*sin(*φ*) at *θ* = *φ*. The latter case is interesting because it represents a point at which both pore types have the same radius and also represents a minimum so that increasing or decreasing *θ* will lead to an increase in pore size, a characteristic which can only be exhibited by Type *β* systems. All this restricts the variability in the pore radius when compared to their respective space filling Type *α* models.

**Figure 4:**
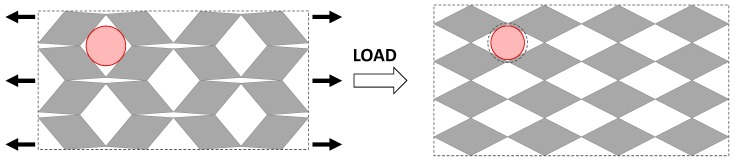
A rotating rhombi network behaving auxetically on stretching while the pore radius decreases.

Type II*β* parallelograms, together with Type *β* rhombi, Type II rectangles and squares offer the most consistent Poisson’s ratios, making them perhaps the most ideal to use for filtration applications. For these systems, the Poisson’s ratio is always negative and fixed at a value of −1, so that filters based on these geometries retain their initial proportions. In this respect, it is important to note that auxeticity does not necessarily imply that the pore size increases as the system is stretched, even though the area of the system increases. In other words, in general, there can be no definite association between a negative Poisson’s ratio and an increase in pore size. In fact, from Table 2, one may note that there are ranges of *θ* over which the Poisson’s ratio is negative and the pore radius decreases on stretching (e.g., Type *β* rhombi and Type II*β* parallelograms). Such behaviour, however, is only possible because of the presence of two different sets of pores which behave opposite to each other, i.e., as the structure is stretched towards its fully opened configuration, one set of pores open up while the others close down in such a way that the maximum pore radius decreases while the system itself expands in all directions (see Figure 4). It is also interesting to note that in the fully open configuration of Type *β* rhombi all pores have the same size so that more particles of that radius can pass through than in any other configuration. This offers a way how to control the rate of flow of particles through the filter allowing it to be set on two modes—a low transmission mode and a high transmission mode.

Before concluding, one should note that the work presented in this section is important not only because it has been confirmed that the auxetic structures described here have excellent potential for use in filtering systems that can be tuned for particular particle sizes, but also because expressions which quantify the performance and characteristics of various filter geometries of this type have been presented. In particular, it has been shown that the Type α systems in general can offer a wider range of pore sizes than the Type *β* counterpart. The advantage of such tunability lies in the fact that such filters need not be dismantled each time the size of the particles that need to be filtered is changed. Here, it should be emphasized that the models presented are only considered from a purely geometric perspective and assume that the filtered medium is a collection of rigid particles. In other words, it does not consider any interactions that may be present between the filtered medium and the porous auxetic structures and neglects properties pertaining to the filtered material such as isochoric deformations, pressure and velocity of the fluid and viscosity. Thus, although the models give an insight vis-à-vis the geometrical aspect of such filters, they may be inadequate to fully describe filtration behaviour of real systems, which may be more complex.

Besides filtration, another interesting application for the use of such systems is in controlling light intensity. Porous structures which are constructed from beam like elements rather than plates, such as hexagonal honeycombs, may not be very suitable for such a purpose because of their high porosity. In contrast, in rotating rigid units systems, the space coverage and therefore the amount of light allowed to pass through can be easily controlled not only by varying *θ* but also by varying parameters *a*, *b* and *φ* and the type of connectivity of the units. Thus, for example, one can use one of the space filling structures to design a blind structure which can either partially or completely obstruct light.

These systems could also be used for skin grafting applications where one could use smaller grafts from donor sites that expand in both directions covering larger areas than grafts having the more typical honeycomb motif. The fact that the rotating rigid units also allow for variability in pore shapes and sizes could also have implications in healing time and extent of scarring.

As a final note, it is important to emphasize that the rotating rigid quadrilateral systems discussed here are meant to complement the re-entrant filters studied by Alderson et al. [85] and Lim et al. [84], where the advantages associated with auxetic filters have already been amply demonstrated. In fact, the systems studied here share some similarities with re-entrant hexagonal honeycombs in the sense that in general, their auxetic behavior can also be used to design filters with tunable porosity and defouling properties. It is beyond the scope of this work to provide a full comparison of these two classes of systems particularly in view of the fact that this and the earlier work complement each other. It is ultimately up to the end user to decide which systems is most amenable for their particular needs as specific practical requirements may necessitate the use of one system rather than the other. For example, if these systems had to be constructed as a membrane with slits, then the systems presented here may lend themselves better as blueprints for their construction. On the other hand if a system with a very high pore space coverage is required, the re-entrant filters might be more suitable than the ones studied here.

## 4. Conclusions

Systems based on rotating rigid units can offer a wide range of pore size and space coverage properties that can be useful in applications such as sieving applications. This work suggests that the range of pore size can be efficiently controlled by using different types of connectivity. In particular, it has been shown that systems derivable from Type *α* parallelograms have a wider range of pore size as opposed to Type *β* systems which could have a much more restricted range. Moreover, this work also highlights the fact that a higher porosity (space coverage) and negative Poisson’s ratios do not necessarily lead to larger pore radius for these systems.

## Figures and Tables

**Table 1:** Comparison of pore shape and size for rotating rigid quadrilateral networks.

Quadrilateral		Pore Shape	Congruent	Similar	Space Filling
Squares	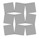	Rhombus	Yes	Yes	Yes
Type I rectangles	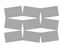	Rhombus	No	Yes	Yes
Type II rectangles	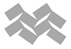	Parallelogram	Yes	Yes	Yes
Type α rhombi	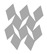	rhombus	Yes	Yes	Yes
Type β rhombi	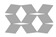	rhombus	No	No	No
Type Iα parallelograms	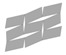	Rhombus	No	Yes	No
Type IIα parallelograms	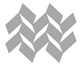	Parallelogram	Yes	Yes	Yes
Type Iβ parallelograms	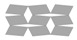	Rhombus	No	No	No
Type IIβ parallelograms	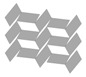	Parallelogram	No	No	No

**Table 2:** Expressions relating space coverage, pore radius (radius of circle inscribed in the pore) and Poisson’s ratios to geometric parameters of the systems for cases where *a* > *b*. The vertical dashed line in the plots of the Poisson ratios represents asymptotic behavior that occurs when the system is locked in the loading direction.

**System**	**Squares [57]**	**Type *α* Rhombi [60]**	**Type *β* Rhombi [61]**
**Parameters of geometries**	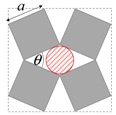	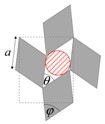	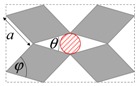
**Space coverage**	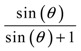	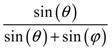	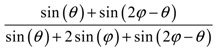
**Pore radius**	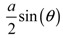	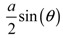	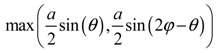
	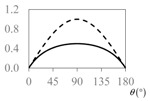	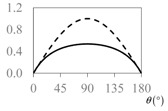	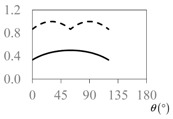
	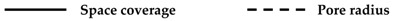		
**Poisson’s ratio (*ν_xy_*)**	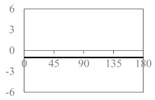	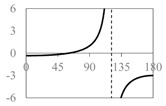	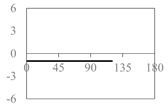
**System**	**Type I Rectangles** [89]	**Type I*α* Parallelograms** [61]	**Type I*β* Parallelograms** [61]
**Parameters of geometries**	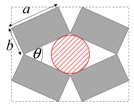	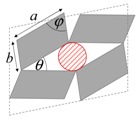	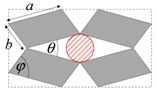
**Space coverage**	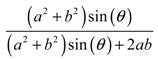	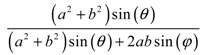	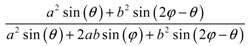
**Pore radius**	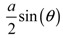	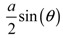	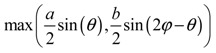
	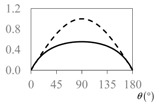	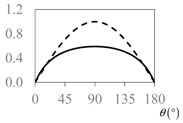	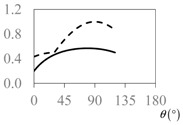
	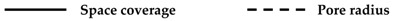		
**Poisson’s ratio (*ν_xy_*)**	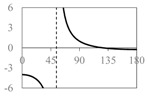	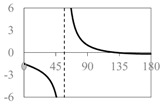	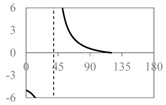
**System**	**Type II Rectangles** [62]	**Type II*α* Parallelograms** [60]	**Type II*β* Parallelograms** [61]
**Parameters of geometries**	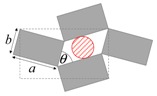	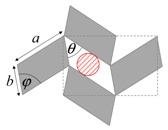	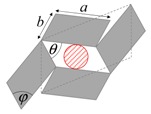
**Space coverage**	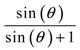	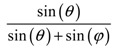	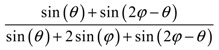
**Pore radius**	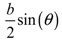	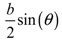	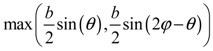
	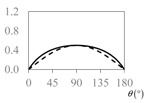	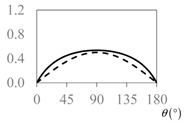	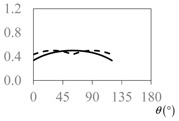
	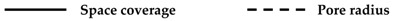		
**Poisson’s ratio (*ν_xy_*)**	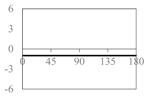	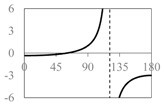	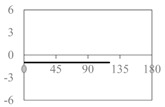

## References

[1] Love A.E.H. (1927). A Treatise on the Mathematical Theory of Elasticity.

[2] Wojciechowski K.W., Brańka A.C. (1989). Negative Poisson ratio in a two-dimensional “‘isotropic’” solid. Phys. Rev. A.

[3] Evans K.E., Nkansah M.A., Hutchinson I.J., Rogers S.C. (1991). Molecular network design. Nature.

[4] Gibson L.J., Ashby M.F., Schajer G.S., Robertson C.I. (1982). The mechanics of two-dimensional cellular materials. Proc. R. Soc. A.

[5] Almgren R.F. (1985). An isotropic three-dimensional structure with Poisson’s ratio-1. J. Elast..

[6] Kolpakov A.G. (1985). Determination of the average characteristics of elastic frameworks. J. Appl. Math. Mech..

[7] Wojciechowski K.W. (1987). Constant thermodynamic tension Monte Carlo studies of elastic properties of a two-dimensional system of hard cyclic hexamers. Mol. Phys..

[8] Popereka M.Y., Balagurov V.G. (1970). Ferromagnetic films having a negative Poisson ratio. Sov. Phys. Solid State.

[9] Milstein F., Huang K. (1979). Existence of a negative Poisson ratio in fcc crystals. Phys. Rev. B.

[10] Lim T.C. (2015). Auxetic Materials and Structures.

[11] Saxena K.K., Das R., Calius E.P. (2016). Three decades of auxetics research—Materials with negative Poisson’s ratio: A review. Adv. Eng. Mater..

[12] Ren X., Das R., Tran P., Ngo T.D., Xie Y.M. (2018). Auxetic metamaterials and structures: A review. Smart Mater. Struct..

[13] Yeganeh-Haeri A., Weidner D.J., Parise J.B. (1992). Elasticity of α-cristobalite: A silicon dioxide with a negative Poisson’s ratio. Science.

[14] Grima J.N., Jackson R., Alderson A., Evans K.E. (2000). Do zeolites have negative Poisson’s ratios?. Adv. Mater..

[15] Sanchez-Valle C., Lethbridge Z.A.D., Sinogeikin S.V., Williams J.J., Walton R.I., Evans K.E., Bass J.D. (2008). Negative Poisson’s ratios in siliceous zeolite MFI-silicalite. J. Chem. Phys..

[16] Azzopardi K.M., Brincat J.P., Grima J.N., Gatt R. (2015). Anomalous elastic properties in stishovite. RSC Adv..

[17] Rovati M. (2004). Directions of auxeticity for monoclinic crystals. Scr. Mater..

[18] Baughman R.H., Shacklette J.M., Zakhidov A.A., Stafström S. (1998). Negative Poisson’s ratios as a common feature of cubic metals. Nature.

[19] Brańka A.C., Heyes D.M., Wojciechowski K.W. (2009). Auxeticity of cubic materials. Phys. Status Solidi.

[20] Krasavin V.V., Krasavin A.V. (2014). Auxetic properties of cubic metal single crystals. Phys. Status Solidi.

[21] Baughman R.H., Galvão D.S. (1993). Crystalline networks with unusual predicted mechanical and thermal properties. Nature.

[22] Grima J.N., Attard D., Cassar R.N., Farrugia L., Trapani L., Gatt R. (2008). On the mechanical properties and auxetic potential of various organic networked polymers. Mol. Simul..

[23] Cadelano E., Palla P.L., Giordano S., Colombo L. (2010). Elastic properties of hydrogenated graphene. Phys. Rev. B.

[24] Grima J.N., Winczewski S., Mizzi L., Grech M.C., Cauchi R., Gatt R., Attard D., Wojciechowski K.W., Rybicki J. (2015). Tailoring graphene to achieve negative Poisson’s ratio properties. Adv. Mater..

[25] Jiang J.-W., Chang T., Guo X., Park H.S. (2016). Intrinsic negative Poisson’s ratio for single-layer graphene. Nano Lett..

[26] Qin H., Sun Y., Liu J.Z., Li M., Liu Y. (2017). Negative Poisson’s ratio in rippled graphene. Nanoscale.

[27] Wojciechowski K.W. (2003). Non-chiral, molecular model of negative Poisson ratio in two dimensions. J. Phys. A. Math. Gen..

[28] Wojciechowski K.W., Tretiakov K.V., Kowalik M. (2003). Elastic properties of dense solid phases of hard cyclic pentamers and heptamers in two dimensions. Phys. Rev. E.

[29] Pigłowski P., Narojczyk J., Poźniak A., Wojciechowski K., Tretiakov K. (2017). Auxeticity of Yukawa Systems with Nanolayers in the (111) Crystallographic Plane. Materials.

[30] Lakes R. (1987). Foam Structures with a negative Poisson’s ratio. Science.

[31] Chan N., Evans K.E. (1997). Fabrication methods for auxetic foams. J. Mater. Sci..

[32] Grima J.N., Attard D., Gatt R., Cassar R.N. (2009). A novel process for the manufacture of auxetic foams and for their re-conversion to conventional form. Adv. Eng. Mater..

[33] Scarpa F., Pastorino P., Garelli A., Patsias S., Ruzzene M. (2005). Auxetic compliant flexible PU foams: Static and dynamic properties. Phys. Status Solidi Basic Res..

[34] Bianchi M., Scarpa F., Banse M., Smith C.W. (2011). Novel generation of auxetic open cell foams for curved and arbitrary shapes. Acta Mater..

[35] Critchley R., Corni I., Wharton J.A., Walsh F.C., Wood R.J.K., Stokes K.R. (2013). A review of the manufacture, mechanical properties and potential applications of auxetic foams. Phys. Status Solidi.

[36] He C., Liu P., Griffin A.C. (1998). Toward negative Poisson ratio polymers through molecular design. Macromolecules.

[37] He C., Liu P., McMullan P.J., Griffin A.C. (2005). Toward molecular auxetics: Main chain liquid crystalline polymers consisting of laterally attached para-quaterphenyls. Phys. Status Solidi.

[38] Alderson K.L., Evans K.E. (1992). The fabrication of microporous polyethylene having a negative Poisson’s ratio. Polymer.

[39] Alderson K.L., Alderson A., Smart G., Simkins V.R., Davies P.J. (2002). Auxetic polypropylene fibres:Part 1—Manufacture and characterisation. Plast. Rubber Compos..

[40] Herakovich C.T. (1984). Composite Laminates with Negative Through-the-Thickness Poisson’s Ratios. J. Compos. Mater..

[41] Evans K.E., Nkansah M.A., Hutchinson I.J. (1992). Modelling negative Poisson ratio effects in network-embedded composites. Acta Metall. Mater..

[42] Milton G.W. (1992). Composite materials with poisson’s ratios close to—1. J. Mech. Phys. Solids.

[43] Alderson K.L., Simkins V.R., Coenen V.L., Davies P.J., Alderson A., Evans K.E. (2005). How to make auxetic fibre reinforced composites. Phys. Status Solidi.

[44] Bezazi A., Boukharouba W., Scarpa F. (2009). Mechanical properties of auxetic carbon/epoxy composites: Static and cyclic fatigue behaviour. Phys. Status Solidi.

[45] Miller W., Hook P.B., Smith C.W., Wang X., Evans K.E. (2009). The manufacture and characterisation of a novel, low modulus, negative Poisson’s ratio composite. Compos. Sci. Technol..

[46] Strek T., Jopek H. (2012). Effective mechanical properties of concentric cylindrical composites with auxetic phase. Phys. Status Solidi.

[47] Strek T., Jopek H., Idczak E., Wojciechowski K. (2017). Computational Modelling of structures with non-intuitive behaviour. Materials.

[48] Jopek H. (2017). Finite Element Analysis of Tunable Composite Tubes Reinforced with Auxetic Structures. Materials.

[49] Jopek H., Stręk T. (2018). Thermoauxetic Behavior of Composite Structures. Materials.

[50] Sloan M.R., Wright J.R., Evans K.E. (2011). The helical auxetic yarn—A novel structure for composites and textiles; geometry, manufacture and mechanical properties. Mech. Mater..

[51] Alderson K.L., Alderson A., Anand S., Simkins V., Nazare S., Ravirala N. (2012). Auxetic warp knit textile structures. Phys. Status Solidi.

[52] Ugbolue S.C., Kim Y.K., Warner S.B., Fan Q., Yang C.L., Kyzymchuk O., Feng Y. (2010). The formation and performance of auxetic textiles. Part I: Theoretical and technical considerations. J. Text. Inst..

[53] Hu H., Wang Z., Liu S. (2011). Development of auxetic fabrics using flat knitting technology. Text. Res. J..

[54] Wright J.R., Burns M.K., James E., Sloan M.R., Evans K.E. (2012). On the design and characterisation of low-stiffness auxetic yarns and fabrics. Text. Res. J..

[55] Glazzard M., Breedon P. (2014). Weft-knitted auxetic textile design. Phys. Status Solidi.

[56] Ge Z., Hu H. (2013). Innovative three-dimensional fabric structure with negative Poisson’s ratio for composite reinforcement. Text. Res. J..

[57] Grima J.N., Evans K.E. (2000). Auxetic behavior from rotating squares. J. Mater. Sci. Lett..

[58] Grima J.N., Evans K.E. (2000). Self expanding molecular networks. Chem. Commun..

[59] Grima J.N., Chetcuti E., Manicaro E., Attard D., Camilleri M., Gatt R., Evans K.E. (2012). On the auxetic properties of generic rotating rigid triangles. Proc. R. Soc. A.

[60] Grima J.N., Farrugia P.S., Gatt R., Attard D. (2008). On the auxetic properties of rotating rhombi and parallelograms: A preliminary investigation. Phys. Status Solidi.

[61] Attard D., Manicaro E., Grima J.N. (2009). On rotating rigid parallelograms and their potential for exhibiting auxetic behaviour. Phys. Status Solidi.

[62] Grima J.N., Gatt R., Alderson A., Evans K.E. (2005). On the auxetic properties of ‘rotating rectangles’ with different connectivity. J. Phys. Soc. Japan.

[63] Evans K.E., Alderson A. (2001). Rotation and dilation deformation mechanisms for auxetic behaviour in the α-cristobalite tetrahedral framework structure. Phys. Chem. Miner..

[64] Attard D., Grima J.N. (2012). A three-dimensional rotating rigid units network exhibiting negative Poisson’s ratios. Phys. Status Solidi.

[65] Shen J., Zhou S., Huang X., Xie Y.M. (2014). Simple cubic three-dimensional auxetic metamaterials. Phys. Status Solidi.

[66] Kim J., Shin D., Yoo D.S., Kim K. (2017). Regularly configured structures with polygonal prisms for three-dimensional auxetic behaviour. Proc. R. Soc. A.

[67] Alderson A., Evans K.E. (2009). Deformation mechanisms leading to auxetic behaviour in the α-cristobalite and α-quartz structures of both silica and germania. J. Phys. Condens. Matter.

[68] Grima J.N., Gatt R., Alderson A., Evans K.E. (2005). On the origin of auxetic behaviour in the silicate α-cristobalite. J. Mater. Chem..

[69] Nazaré F., Alderson A. (2015). Models for the prediction of Poisson’s ratio in the “α-cristobalite” tetrahedral framework. Phys. Status Solidi.

[70] Alderson A., Evans K.E. (2002). Molecular origin of auxetic behavior in tetrahedral framework silicates. Phys. Rev. Lett..

[71] Grima J.N., Gatt R., Zammit V., Williams J.J., Evans K.E., Alderson A., Walton R.I. (2007). Natrolite: A zeolite with negative Poisson’s ratios. J. Appl. Phys..

[72] Grima J.N., Alderson A., Evans K.E. (2005). An alternative explanation for the negative Poisson’s ratios in auxetic foams. J. Phys. Soc. Japan.

[73] McDonald S.A., Dedreuil-Monet G., Yao Y.T., Alderson A., Withers P.J. (2011). In situ 3D X-ray microtomography study comparing auxetic and non-auxetic polymeric foams under tension. Phys. status solidi.

[74] Ali M.N., Rehman I.U. (2011). An Auxetic structure configured as oesophageal stent with potential to be used for palliative treatment of oesophageal cancer; development and in vitro mechanical analysis. J. Mater. Sci. Mater. Med..

[75] Ali M.N., Busfield J.J.C., Rehman I.U. (2014). Auxetic oesophageal stents: Structure and mechanical properties. J. Mater. Sci. Mater. Med..

[76] Cross T.M., Hoffer K.W., Jones D.P., Kirschner P.B., Langvin E., Meschter J.C. (2016). Auxetic Structures and Footwear with Soles having Auxetic Structures. U.S. Patent.

[77] Blakely K.S., Toronjo A. (2013). Articles of Apparel with Auxetic Fabric. U.S. Patent.

[78] Konakovi M., Keenan E., Cmu C., Deng B., Bouaziz S., Piker D., Epfl M.P. (2016). Beyond developable: Computational design and fabrication with auxetic materials. ACM Trans. Graph. TOG.

[79] Tang Y., Yin J. (2017). Design of cut unit geometry in hierarchical kirigami-based auxetic metamaterials for high stretchability and compressibility. Extrem. Mech. Lett..

[80] Grima J.N., Gatt R. (2010). Perforated sheets exhibiting negative Poisson’s ratios. Adv. Eng. Mater..

[81] Hou X., Hu H., Silberschmidt V. (2012). A novel concept to develop composite structures with isotropic negative Poisson’s ratio: Effects of random inclusions. Compos. Sci. Technol..

[82] Poźniak A.A., Wojciechowski K.W., Grima J.N., Mizzi L. (2016). Planar auxeticity from elliptic inclusions. Compos. Part B Eng..

[83] Grima J.N., Alderson A., Evans K.E. (2004). Negative Poisson’s ratios from rotating rectangles. Comput. Methods Sci. Technol..

[84] Lim T.C., Acharya R.U. (2010). Performance evaluation of auxetic molecular sieves with re-entrant structures. J. Biomed. Nanotechnol..

[85] Alderson A., Rasburn J., Ameer-Beg S., Mullarkey P.G., Perrie W., Evans K.E. (2000). An auxetic filter: A tuneable filter displaying enhanced size selectivity or defouling properties. Ind. Eng. Chem. Res..

[86] Alderson A., Rasburn J., Evans K.E., Grima J.N. (2001). Auxetic polymeric filters display enhanced de-fouling and pressure compensation properties. Membr. Technol..

[87] Rasburn J., Mullarkey P.G., Evans K.E., Alderson A., Ameer-Beg S., Perrie W. (2001). Auxetic structures for variable permeability systems. AIChE J..

[88] Alderson A., Rasburn J., Evans K.E. (2007). Mass transport properties of auxetic (negative Poisson’s ratio) foams. Phys. Status Solidi.

[89] Grima J.N., Alderson A., Evans K.E. (2005). Auxetic behaviour from rotating rigid units. Phys. Status Solidi.

